# Safety of Percutaneous Muscle Biopsy: An Update Based on Over 2400 Procedures

**DOI:** 10.1111/sms.70160

**Published:** 2025-11-03

**Authors:** Jhonnatan Vasconcelos Pereira Santos, Andresa Rossilho Casale, Rafael de Almeida Azevedo, Gabriela Borelli Garcia Eluf Politi, Samuel Katsuyuki Shinjo, Hamilton Roschel, Bruno Gualano

**Affiliations:** ^1^ Applied Physiology and Nutrition Research Group—School of Physical Education and Sport, Faculdade de Medicina FMUSP Universidade de São Paulo São Paulo SP Brazil; ^2^ Center of Lifestyle Medicine, Faculdade de Medicina FMUSP Universidade de São Paulo São Paulo SP Brazil; ^3^ Rheumatology Division, Faculdade de Medicina FMUSP Universidade de São Paulo São Paulo SP Brazil; ^4^ Laboratory of Assessment and Conditioning in Rheumatology, Hospital das Clínicas HCFMUSP, Faculdade de Medicina Universidade de São Paulo São Paulo SP Brazil

**Keywords:** adverse events, muscle biopsy, skeletal muscle

## Abstract

This study assessed the safety of percutaneous muscle biopsy by analyzing over 2400 procedures performed between 2007 and 2025 in healthy individuals and patients with chronic diseases. We retrospectively reviewed biopsy‐related adverse events among 1246 participants (471 healthy adults and 775 patients) to investigate whether health status or repeated sampling at the same site influenced complication rates. A total of 2435 biopsies were performed, primarily on the vastus lateralis muscle (97%). Overall, 80.6% of the procedures had no adverse events. The most common adverse events were erythema (6.6%), ecchymosis (4.1%), and pain (3.9%). Serious complications, including loss of consciousness (vasovagal syncope), were extremely rare (≤ 0.04%). Most adverse events occurred in isolation (74.9%) and did not preclude participation in subsequent research activities. Repeated biopsies modestly increased the risk of minor adverse events, particularly bleeding, erythema, and bruising. Notably, these were more frequently reported among healthy volunteers than among patients. In conclusion, percutaneous muscle biopsy is a safe and well‐tolerated procedure, even when performed repeatedly or in individuals with chronic diseases. The minor adverse events were infrequent, transient, and clinically manageable. These findings support the continued use of this technique in both research and clinical settings.

## 1 Introduction

Percutaneous muscle biopsy has evolved significantly since it was first introduced in the 1960s [1, 2]. It is a minimally invasive technique for collecting skeletal muscle tissue in vivo, involving a straightforward clinical procedure that ensures a high sample yield in a time‐efficient manner. Muscle tissue analysis serves multiple purposes, from clinical diagnosis to the study of the physiological mechanisms underlying pharmacological and non‐pharmacological interventions, such as exercise and nutrition. Given its broad utility in both research and clinical settings, understanding the safety profile of muscle biopsies is essential.

Our group previously examined the incidence of adverse events related to percutaneous muscle biopsy in 274 volunteers (496 procedures), reporting outcomes in healthy individuals and patients with chronic diseases [3]. Pain, erythema, and ecchymosis were the most frequently reported adverse events, affecting 1.27% of the participants. Less commonly, panic episodes (0.21%), bleeding (0.42%), and edema (0.84%) were also reported. Although several retrospective analyses have similarly reported a low incidence of adverse events [4, 5], the circumstances under which such events occur remain poorly understood.

The safety of needle muscle biopsy has been extensively documented, as Tarnopolsky et al. [6] reported a relatively low adverse event rate (0.15%) among patients undergoing 13 500 procedures. However, it remains uncertain whether the safety of muscle biopsies differs between healthy individuals and patients, nor whether repeated biopsies, particularly at the same muscle site, increase the risk of adverse events. These questions are particularly relevant given the growing use of serial muscle biopsies in both clinical and research settings. Addressing such gaps is essential for refining biopsy protocols and ensuring participant safety.

This study provides an updated analysis of biopsy‐related adverse events, building upon our earlier findings [3] by exploring whether adverse events differ based on participants' health status and examining the potential cumulative effects of repeated biopsies on the same muscle.

## 2 Materials and Methods

This study is a retrospective analysis of the incidence of adverse events associated with percutaneous muscle biopsy. A systematic and active search for adverse events was conducted using the medical records of volunteers enrolled in cross‐sectional and longitudinal studies at our institution's research sites.

The sample included adult healthy individuals and patients with chronic diseases, with the number of biopsies per participant ranging from one to eight, depending on the objectives and design of each original study. All participants provided written informed consent, and all original studies were approved by the Ethics Committee of our institution, following international ethical standards.

### 2.1 Percutaneous Muscle Biopsies

All percutaneous muscle biopsies followed a standardized protocol [7] and were performed by a medical doctor (MD) with the assistance of a skilled collaborator (graduate student). Importantly, all personnel involved in the biopsy procedures underwent a standardized training procedure before participating in the studies included herein. The biopsy protocol started by cleaning and disinfecting the skin over the muscle surface thoroughly using an iodophor‐based antiseptic solution. Lidocaine (2%) was infiltrated into the skin and subcutaneous tissue to induce local anesthesia. Subsequently, an incision (0.5 cm) was made through these layers at the designated biopsy site on the (i) vastus lateralis, 25‐cm proximal to the tibial tuberosity, and 5‐cm lateral to the midline of the femoral axis; (ii) deltoid, at its most lateral and voluminous portion [8]; and (iii) medial portion of the gastrocnemius muscle of the right leg [9]. A 5‐mm modified Bergström needle was then inserted through the fascia, and an assistant immediately applied suction using a 60‐mL syringe connected to a vacuum canister mounted atop the needle. Muscle tissue was aspirated, and the incision was closed using skin closure strips (Steri‐Strip 3M). A transparent film dressing (TegaderFilm 3M) was used to cover the biopsy site. To minimize the risk of infection and bruising, a pressure dressing was applied immediately and maintained for 72 h.

The aim of each biopsy insertion was to obtain at least 50 mg of muscle tissue. Whenever the sample was insufficient, a new insertion was performed upon patient consent, using the same incision site [10]. In cases of repeated biopsies within the same experimental session, a single incision was reused. In cases of repeated biopsies on separate days, new incisions were performed, ranging from 0.5 (early studies from the laboratory) [3] to 3 cm distance between incisions (more recent studies; added dataset since previous publication). In case of reinsertion using the same incision, the second sample was obtained by introducing the biopsy needle at a different angle to the muscle belly than the previous biopsy.

### 2.2 Sterilization Protocol

All biopsy needles were used brand new whenever available. For reusable instruments, a strict sterilization protocol was followed. First, after use, needles were immediately immersed in a solution of enzymatic detergent prepared with distilled water. The solution was injected into the lumen of the needle several times, and the instrument remained submerged for a minimum of 5 min. Second, needles were disassembled and placed in an ultrasonic bath with distilled water and enzymatic detergent. The ultrasonic cycle was run for 8 min at 65°C, and additional cycles were performed if necessary to ensure thorough cleaning. Then, instruments were rinsed thoroughly with running water, instilling water under pressure through the lumen, and subsequently submerged in distilled water for at least 2 h. Next, needles were dried externally and internally with clean absorbent material, visually inspected for residual debris, and placed in an oven for complete drying. Finally, each instrument was packaged individually with its corresponding number and sterilized by autoclaving prior to use [11].

### 2.3 Adverse Events

Any biopsy‐related events were recorded during the procedure. In addition, all participants received standardized post‐biopsy care instructions and were advised to promptly report any complications to the medical or research team during follow‐up. Specifically, participants were informed that mild local pain is common and usually subsides spontaneously. In cases of moderate and intense local pain, the use of over‐the‐counter analgesics was permitted. On the day following the procedure, participants were instructed to apply an ice pack to the biopsy site three times a day for 15 min and to avoid local heat exposure. The dressing was to remain dry and intact for 72 h; after this period, participants were instructed to remove it themselves and clean the site gently with water and soap, followed by a drying procedure without rubbing. Physical activity was unadvised during the first 48 h post‐biopsy, and strenuous exercise was discouraged during the first week after the procedure. In addition, participants were advised to protect any ecchymosis from sun exposure to prevent hyperpigmentation. Finally, they were instructed to immediately contact the research or medical team in case of any bleeding, discharge, redness, swelling, persistent pain, or any other concerning symptoms.

Data collection for this study was guided by a standardized list of 15 predefined adverse events, including bleeding, ecchymosis, edema, erythema, hematoma, inflammation, keloid formation, local atrophy, local infection, loss of consciousness, denervation, nonhealing, numbness, pain, and panic attacks, with the possibility of reporting additional events not captured by this list. Factors that may contribute to the risk of adverse events, such as repeated procedures at the same site and the use of antithrombotic agents, were documented.

### 2.4 Statistical Analyses

A descriptive analysis was conducted to explore the incidence of adverse events. Data are presented as absolute frequencies, proportions, and their corresponding 95% confidence intervals (95% CI), estimated using the Clopper–Pearson exact method. The incidence and relative risk (RR) of each adverse event were estimated with 95% CI to compare health status (healthy vs. patients) and procedure repetition (1st vs. ≥ 2nd biopsies). Data collected between 2010 and 2025 were organized in a Microsoft Excel spreadsheet, and all analyses were performed using the jamovi statistical software (version 2.6). A significance level (*α*) of 0.050 was used.

## 3 Results

**FIGURE 1:**
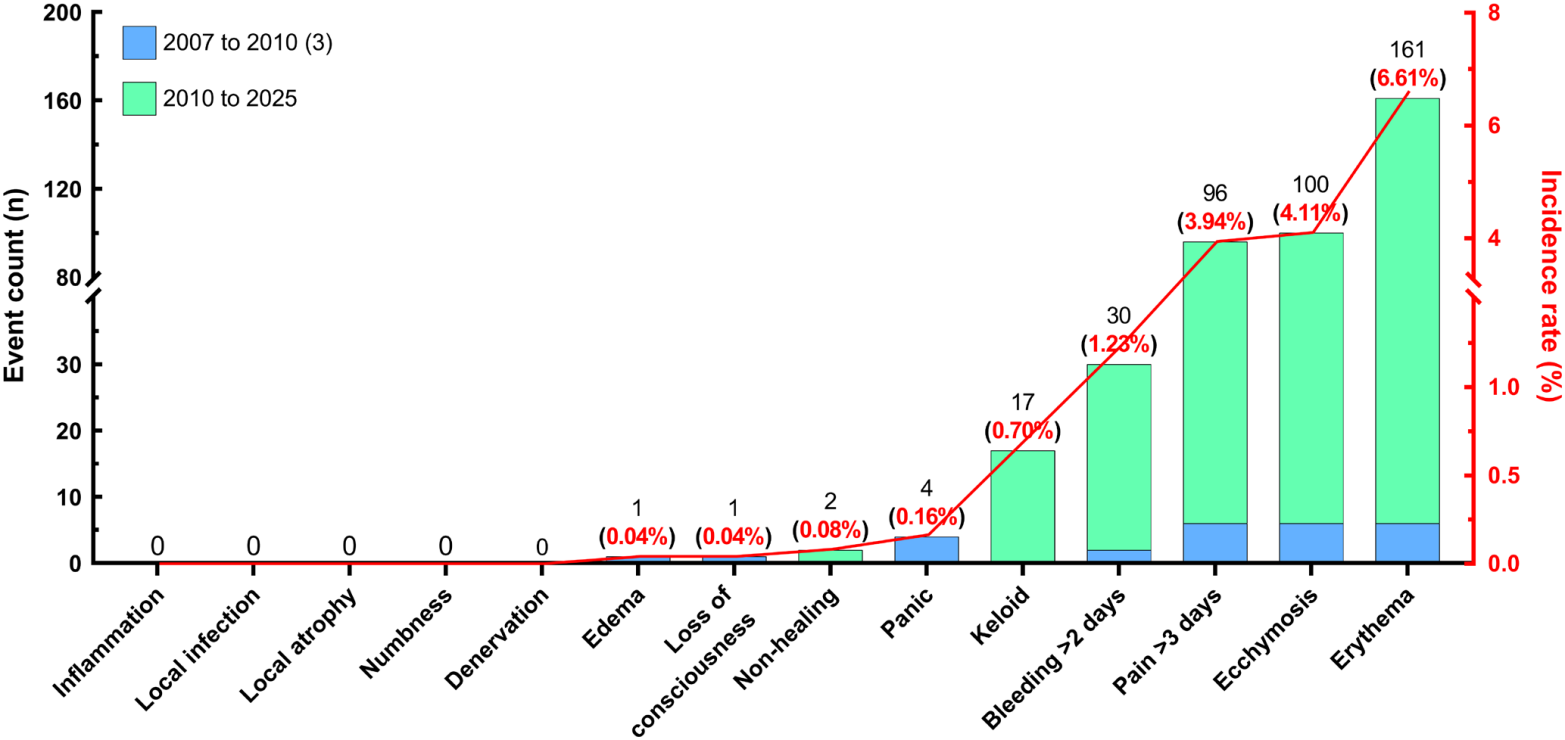
Percutaneous muscle biopsy‐related adverse events.

Medical records of 1246 research volunteers, including 471 healthy individuals and 775 patients with chronic diseases, who underwent a total of 2435 percutaneous muscle biopsies between 2007 and 2025 were retrospectively reviewed. Adverse events occurred in 19.4% of the procedures; however, in 74.9% of these cases, only a single event was reported, indicating that complications were typically isolated. Nine distinct adverse events were identified, with incidence rates ranging from 0.04% (edema) to 6.6% (erythema). These events included local vasculoinflammatory reactions (erythema, edema, and ecchymosis), persistent post‐procedural pain, bleeding, wound‐related outcomes (nonhealing and keloid formation), and transient systemic responses, such as loss of consciousness and acute panic episodes. Clinically significant adverse events, such as loss of consciousness (vasovagal syncope with spontaneous recovery within 5 s), were rare, occurring in only a single isolated procedure each (≤ 0.04%) (Figure 1).

Table 1 presents the characteristics of the sample stratified by health status. Since 2010, a median of two percutaneous muscle biopsies was performed per subject in our studies, most of which were obtained from the vastus lateralis muscle (97.4%). The patient group included individuals with a range of primary diagnoses, such as muscular dystrophies, obesity, and other chronic conditions. Among the patients with rheumatoid arthritis, only one was receiving antithrombotic therapy (ticlopidine 25 mg every 12 h, initiated on August 8, 2017, for secondary prevention of possible cerebral ischemia), and underwent four muscle biopsies (i.e., two in the same session, on 11/01/2018, and the remaining on 11/08/2018 and 11/22/2018). The medication was continued in all procedures, without experiencing any adverse events. Compared with healthy volunteers, the patient group was older, more often female, and had a higher body mass index (*p* < 0.001).

**TABLE 1:** Participant demographic characteristics by health status (2010–2025).

	Patients (*n* = 669)	Healthy (*n* = 303)	Full sample (*n* = 972)
Age, years	46.49 ± 14.43	27.38 ± 6.60	40.82 ± 15.35
Sex			
Male	211 (31.54)	268 (88.45)	479 (49.28)
Female	458 (68.46)	35 (11.55)	493 (50.72)
BMI, kg.m^−2^	33.02 ± 11.12	24.59 ± 3.23	28.26 ± 8.78
Antithrombotic therapy	1 (0.15)	0 (0.00)	1 (0.10)
Muscle biopsies	1124	1311	2435
Biopsies by subject	1 [1]	3 [2]	1 [1]
Muscle biopsy site			
Vastus lateralis	1092 (97.15)	1281 (97.71)	2.373 (97.45)
Medial gastrocnemius	0 (0.00)	30 (2.29)	30 (1.23)
Deltoid	32 (2.85)	0 (0.00)	32 (1.31)
Main diagnoses			
Muscular dystrophies	207 (30.94)		
Myopathies	143 (21.38)		
Polymyositis	76 (11.36)		
Obesity	53 (7.92)		
Anti‐synthetase syndrome	42 (6.28)		
Paraplegia	32 (4.78)		
Chronic kidney disease	27 (4.04)		
Mild cognitive impairment	25 (3.74)		
Rheumatoid arthritis	18 (2.69)		
Fat liver disease	16 (2.39)		
Polycystic ovary syndrome	15 (2.24)		
MCTD	8 (1.20)		
Long COVID	3 (0.45)		
HyperCKemia	3 (0.45)		
Pompe disease	1 (0.15)		

*Note:* Data are expressed as mean ± standard deviation, median [interquartile range], or frequency (%). Data of muscle biopsy site are expressed as the total number of biopsies made (%).

Abbreviations: BMI, body mass index; COVID, coronavirus disease; MCTD, mixed connective tissue disease.

**FIGURE 2:**
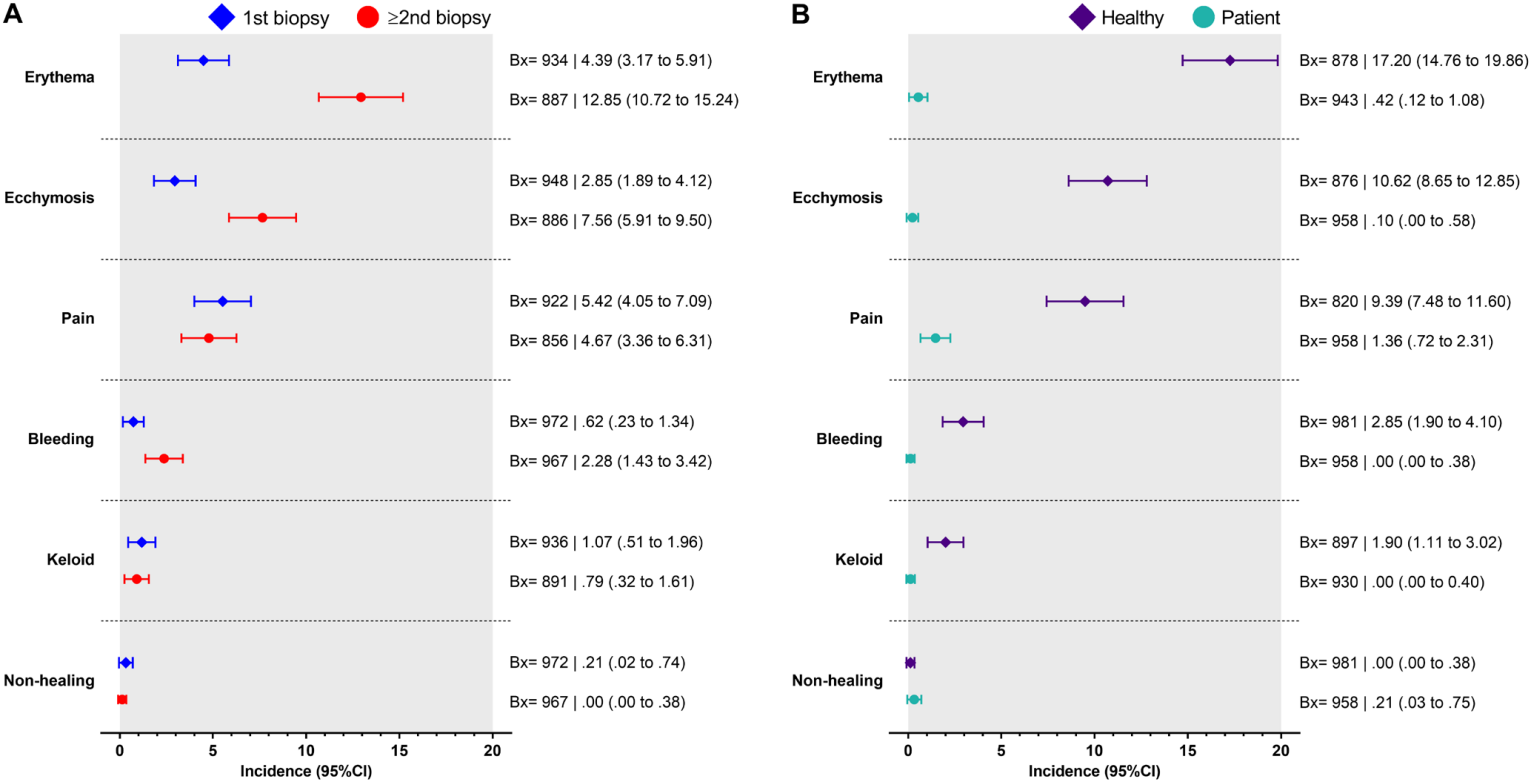
Adverse events by procedure recurrence (1st vs. ≥ 2nd biopsies) (Panel A) and clinical condition (healthy individuals vs. patients with chronic diseases) (Panel B).

Clinical research protocols involving patients resulted in a few adverse events, including pain (13 cases, 1.4%), erythema (four cases, 0.4%), and nonhealing (two cases, 0.2%). Additional analyses revealed that the risk of erythema, ecchymosis, and bleeding was higher in subjects with multiple biopsies when compared to those with a single biopsy (Table 2). Risk for erythema, ecchymosis, and pain was also higher among healthy individuals when compared with patients (Table 2). Erythema, ecchymosis, pain, bleeding, and keloid formation were also more frequent among healthy volunteers (Figure 2). None of these events resulted in additional complications or limited the participant's involvement in the study procedures. Moreover, no cases of local infection, muscle atrophy, denervation, numbness, or panic attacks were recorded. Regarding the adverse events divided by muscle groups, there were 339 for vastus lateralis (erythema *n* = 125, ecchymosis *n* = 94, pain *n* = 90, bleeding *n* = 28, nonhealing *n* = 2), 30 for medial gastrocnemius (all erythema) and none for deltoid. Overall, approximately 96% of the biopsy procedures were conducted by two MDs (out of a total of eight), that underwent the same standardized training procedure before any participation in the studies.

**TABLE 2:** Relative risk of adverse events by health status and procedure repetition.

	Health status			Procedure repetition		
	Healthy^a^	Patient	RR (95% CI)	≥ 2nd biopsy^a^	1st biopsy	RR (95% CI)
Erythema (Bx = 1821)	151/878 (17.20)	4/943 (0.42)	40.54 (15.08–108.96)	114/887 (12.85)	41/934 (4.39)	2.928 (2.07–4.13)
Ecchymosis (Bx = 1834)	93/876 (10.62)	1/958 (0.10)	101.70 (14.20–728.08)	67/886 (7.56)	27/948 (2.85)	2.65 (1.71–4.11)
Pain (Bx = 1778)	77/820 (9.39)	13/958 (1.36)	6.92 (3.87–12.36)	40/856 (4.67)	50/922 (5.42)	0.86 (0.57–1.29)
Bleeding (Bx = 1939)	28/981 (2.85)	0/958 (0.00)	NE	22/967 (2.28)	6/972 (0.62)	3.68 (1.50–9.05)
Keloid (Bx = 1827)	17/897 (1.90)	0/930 (0.00)	NE	7/891 (0.79)	10/936 (1.07)	0.73 (0.28–1.92)
Non‐healing (Bx = 1939)	0/981 (0.00)	2/958 (0.21)	NE	0/967 (0.00)	2/972 (0.21)	NE

*Note:* Data are expressed as the number of adverse events per number of biopsies performed, with corresponding percentages. RR (95% CI), relative risk, and 95% confidence interval.

Abbreviation: NE, not estimable.

^a^ Reference group.

## 4 Discussion

This study updated our previous analysis of adverse events associated with muscle biopsies using a substantially larger dataset collected. We also analyzed whether complication rates differed based on participants' health status and examined the potential cumulative effects of repeated biopsies on the same muscle site. Notably, 81% of the procedures were performed without any adverse events, and no serious complications were reported across the entire cohort, reinforcing the overall safety and tolerability of the technique.

Erythema, ecchymosis, and pain were the most frequently reported adverse events following muscle biopsy, whereas loss of consciousness, nonhealing, and edema were extremely rare.

Performing multiple biopsies on the same muscle site appeared to increase the occurrence of transient skin‐related adverse events, such as erythema, ecchymosis, and less frequently, bleeding. These events, along with keloid formation and pain, were more frequently observed in healthy individuals, and no other complications were reported. Most of the events (nearly 75%) occurred in isolation. Taken together, these findings reinforce the notion that percutaneous muscle biopsies are safe and generally well‐tolerated, even when repeated multiple times within the same participant.

Previous studies have also documented a low occurrence of adverse events following muscle biopsy. For instance, a retrospective analysis based on 13 500 clinical records [6] reported a low incidence of postprocedural complications, including skin infections (0.06%), numbness (0.04%), pain (0.04%), bleeding (0.01%), and ecchymosis (0.01%). Additional outcomes have been documented in smaller‐scale studies, including erythema (1.21%), hyperesthesia (0.07%), panic attacks (0.81%), edema (0.20%), and syncope (0.20%–0.35%) [3, 5, 12, 13]. Our clinical data are consistent with these findings, as only a small proportion (0.10%–1.36%) of skin‐related events, such as erythema and pain, was observed among patients over the past 17 years. During this period, no cases of local infection, muscle atrophy, denervation, numbness, or panic attacks were recorded. The low incidence of adverse events observed in our study is in line with the broader literature, which consistently reports that suction needle muscle biopsy is a safe and well‐tolerated procedure when performed under appropriate conditions [4, 5, 6].

Notably, some adverse events were more frequently observed among healthy volunteers, particularly erythema (17.2%), bruising (10.6%), pain (9.4%), bleeding (2.85%), and keloid formation (1.9%). Although lower rates have been reported in the literature, the experimental nature of some applied physiological protocols may partly account for the higher incidence observed in our setting. For example, one of the included studies had multiple biopsies of the same participant. De Oliveira et al. [14] conducted a crossover trial involving trained cyclists to investigate the ergogenic and metabolic effects of carbohydrate supplementation. Each participant underwent eight muscle biopsies of the vastus lateralis, one before and one after four experimental sessions that included a 2‐h cycling protocol (120 min of vigorous cycling exercise). Notably, one volunteer experienced bleeding following each of the eight biopsy procedures, whereas erythema was a common finding among the participants after the second biopsy on each test day.

Other studies included in our sample also featured intensive experimental protocols that may have contributed to the occurrence of adverse events. Overall, healthy volunteers underwent a greater number of biopsies per subject compared to patients (3.25 vs. 1.46, *p* < 0.001), which may be one of the factors behind the higher incidence of minor complications in this group. In addition, protocols involving multiple same‐site muscle biopsies within a single day, particularly those performed after vigorous exercise, appear to increase the likelihood of vascular and cutaneous adverse events.

Finally, adverse events may differ across anatomical sites. We were unable to test this hypothesis, as only ~2.6% of biopsies were performed in muscles other than the vastus lateralis. Notably, all medial gastrocnemius biopsies were associated with erythema; however, these data come from a single study and may instead reflect the MD's limited experience with this specific muscle site.

These observations highlight the need for future studies to clarify how protocol design and repeated percutaneous muscle biopsies contribute to the occurrence of adverse events.

## 5 Perspective

Whereas this study reinforces the safety of muscle biopsies, there are unexplored questions. First, a standardized adverse events follow‐up protocol should be developed, since the current studies on the topic adopted different time frames and lists of possible adverse events. Based on data from our study and others [6, 7, 15], it is reasonable to propose that individuals undergoing muscle biopsies should be monitored until full resolution of any procedure‐related symptom/sign as duration may persist for weeks or months. Second, future studies could explore the safety and applicability of microbiopsy [16], which might be more suitable for smaller muscles (e.g., tibialis anterior), different populations, such as adolescents, elite athletes or patients with low muscle mass. Third, the utility of ultrasound guidance to improve accuracy, reduce tissue trauma, and minimize neurovascular risk should also be considered, especially in cases of lower muscle sizes and in more severe clinical conditions [17]. Fourth, the impact of MD experience and training on adverse effects remains unclear. This was not analyzed in our study because ~96% of procedures were performed by two MDs who had the same standardized training; future studies should investigate this.

## Conflicts of Interest

The authors declare no conflicts of interest.

## Data Availability

Source data for this study are not publicly available due to privacy or ethical restrictions. The source data are available to verified researchers upon request by contacting the corresponding author.
